# Molecular subtyping of CD5+ diffuse large B-cell lymphoma based on DNA-targeted sequencing and Lymph2Cx

**DOI:** 10.3389/fonc.2022.941347

**Published:** 2022-08-23

**Authors:** Dongshen Ma, Yuhan Ma, Yuanyuan Ma, Jia Liu, Ying Gu, Nian Liu, Chenxi Xiang, Hui Liu, Wei Sang

**Affiliations:** ^1^ Department of Pathology, The Affiliated Hospital of Xuzhou Medical University, Xuzhou, China; ^2^ Department of Hematology, The Affiliated Hospital of Xuzhou Medical University, Xuzhou, China; ^3^ Department of Pathology, Central Hospital Affiliated to Shandong First Medical University, Jinan, China; ^4^ Department of Pathology, Xuzhou Medical University, Xuzhou, China; ^5^ Blood Diseases Institute, Xuzhou Medical University, Xuzhou, China

**Keywords:** CD5, diffuse large B-cell lymphoma, DNA sequencing, genomic profiling, Lymph2Cx

## Abstract

**Background:**

CD5-positive diffuse large B-cell lymphoma (CD5+ DLBCL) showed poor prognosis in the rituximab era, with limited research on its genetic characteristics and cell of origin (COO). We aimed to demonstrate the molecular characteristics of CD5+ DLBCL and to discover potential prognostic factors.

**Methods:**

We included 24 cases of CD5+ DLBCL and 23 CD5-negative (CD5-) counterparts and collected their clinicopathological features. Targeted DNA sequencing of 475 lymphoma-related genes was performed, and all cases were assigned to distinct genetic subtypes using the LymphGen tool. The COO was determined by the Lymph2Cx assay. The Kaplan–Meier method and Cox proportional hazards model were applied to identify the possible prognostic factors.

**Results:**

Compared with their CD5- counterparts, patients with CD5+ DLBCL tended to have a worse prognosis and a higher incidence of MYD88L265P and CD79B double mutation (MCD) subtype (54.17%, P = 0.005) and activated B cell-like (ABC) subtype (62.5%, P = 00017), as determined by next-generation sequencing and Lymph2Cx, respectively. Moreover, PIM1, MYD88, and KMT2D mutations were detected more frequently in CD5+ DLBCL cases (P < 0.05). According to multivariate analysis, MYC/BCL2 double expression and ABC subtype were correlated with unfavorable overall survival (OS). High mRNA expression of SERPINA9 and MME showed a significant correlation with a better OS, and high expression of MME showed a significant correlation with better progression-free survival in CD5+ DLBCL.

**Conclusion:**

The genetic profile of CD5+ DLBCL is characterized by PIM1, MYD88, and KMT2D mutations, with a higher incidence of MCD and ABC subtypes. MYC/BCL2 double expression, ABC subtype, and mRNA expression of SERPINA9 and MME are independently predictive of the prognosis of CD5+ DLBCL.

## Introduction

Diffuse large B-cell lymphoma (DLBCL), the most common B-cell lymphoma, is a heterogeneous group of diseases with distinct clinical, pathological, and genetic characteristics (1). Despite accounting for only 5%–10% of all DLBCL cases, CD5+ DLBCL tends to be more clinically aggressive and have a poorer outcome (2, 3). Compared with CD5-negative (CD5-) DLBCL, CD5+ DLBCL is more common in elderly patients and often shows extranodal involvement (4, 5). More importantly, patients with CD5+ DLBCL do not benefit from rituximab-based immunochemotherapy or stem cell transplantation, with an overall survival (OS) of <30 months when treated with R-EPOCH (rituximab, etoposide, prednisone, vincristine, cyclophosphamide, and doxorubicin) (6, 7). Therefore, it is imperative to explore new therapeutic strategies for CD5+ DLBCL.

Early studies suggested that DLBCL with specific immunophenotypes and genetic mutations showed poor prognosis. Indeed, patients with DLBCL with MYC/BCL2 expression had a lower survival rate than those without double expression (8). *TP53* mutation in DLBCL also has been confirmed to be a strongly unfavorable prognostic factor (9). Recently, the development of classifiers based on gene expression profiling (GEP) and genetic sequencing has revealed the heterogeneity of DLBCL. As a result of GEP, DLBCL cases were classified into three cell-of-origin (COO) categories, including activated B cell–like (ABC; accounting for 30%–40%), germinal center B cell–like (GCB; accounting for 50%–60%), and unclassified (accounting for 10%–20%) (10–12). Among them, ABC DLBCL has an inferior OS than that of GCB DLBCL. Recently, the Lymph2Cx assay has been used to determine the mRNA level of 15 COO-related genes from formalin-fixed paraffin-embedded (FFPE) tissues on the NanoString platform, which has since promoted the clinical use of COO classification (13).

In addition to RNA-based COO classification, genomic studies based on DNA sequencing and fluorescence *in situ* hybridization (FISH) have subclassified DLBCL by genetic variations; these results have confirmed that those molecular classification systems can be used to predict the prognosis of patients and guide treatment choice (14–17). In the well-recognized report published by Schmitz et al. (14) in 2018, DLBCL cases were subclassified into the following five categories: MCD (based on the co-occurrence of *MYD88^L265P^* and *CD79B* mutations), BN2 (based on *BCL6* fusions and *NOTCH2* mutations), EZB (based on *EZH2* mutations and *BCL2* translocations), N1 (based on *NOTCH1* mutations), and “Other” subtype (no specific genetic characteristics). Patients with MCD and N1 subtypes (accounting for 8% and 2.1% of all DLBCL cases, respectively) showed comparatively unfavorable prognosis, whereas those with BN2 and EZB subtypes (accounting for 14.8% and 21.8% of all DLBCL cases, respectively) had a better prognosis (14). These findings provide an accessible methodology and deepen the understanding as to the genetic subtyping of DLBCL. Moreover, the online tool LymphGen offers an accessible algorithm and interface for the practical use of Schmitz’s subtyping (15, 18).

As suggested in recent studies, genetic subtyping-guided R-CHOP (rituximab, cyclophosphamide, doxorubicin, vincristine, and prednisone) regimens and the addition of various drugs [i.e., Bruton’s tyrosine kinase inhibitors (BTKi), lenalidomide] to R-CHOP (termed R-CHOP+X) have improved the outcome of DLBCL (19, 20). Moreover, the COO has been demonstrated to play an important role in the treatment decision. Indeed, ABC DLBCL has been shown to be more responsive to ibrutinib, an inhibitor of B-cell receptor-dependent nuclear factor κB (NF-κB) (21). Therefore, it is necessary to further investigate the genetic characteristics and COO of CD5+ DLBCL to explore the individualized treatment options.

Although it was discovered in early studies that CD5+ DLBCL cases were mainly ABC subtype (22), implying that CD5+ DLBCLs may respond to therapy targeted on ABC DLBCL (23), the pathological and genetic characteristics of CD5+ DLBCL are not yet fully understood, which has hampered the investigation into targeted therapies. In our previous study, several clinicopathological variables were identified, as independent predictors of CD5+ DLBCL, including age, International Prognostic Index (IPI), and MYC expression (24). However, no previous study has examined the genetic variations and COO of CD5+ DLBCL.

In this retrospective study, the genetic and COO characteristics of CD5+ and CD5- DLBCL were investigated by GEP and the LymphGen algorithm, with the aim to identify the molecular factors associated with prognosis and to develop personalized treatment.

## Materials and methods

### Patient selection

We included 24 cases of CD5+ DLBCL diagnosed from 2014 to 2018 at the Affiliated Hospital of Xuzhou Medical University and selected 23 cases of CD5- DLBCL adjusted for age and sex during the same period as the control group. All samples used in this study were from excisional biopsy. Thirteen of 24 CD5+ and 11 of 23 CD5- samples were from lymph nodes. Eleven of 24 CD5+ and 12 of 23 CD5- samples were from extranodal sites, including nasopharynx, tonsil, colon, kidney, and spinal cord. All cases were independently reviewed by pathologists majoring in hematopathology before a consented diagnosis was made according to the World Health Organization (WHO) 2017 guidelines. All cases were confirmed to be CyclinD1-negative [by immunohistochemistry (IHC) and FISH] and SOX11-negative to rule out mantle cell lymphoma (MCL). All cases were confirmed Epstein–Barr–encoding region (EBER)-negative and human immunodeficiency virus (HIV)-negative without any history of chronic lymphocytic leukemia/small lymphocytic lymphoma (CLL/SLL). The pathological and clinical information was retrieved from archives. Follow-up was performed for the patients in December 2018. Informed consent was waived by the investigative review board at our institution because of the retrospective nature of the study.

### Fluorescence *in situ* hybridization and immunohistochemistry

We performed FISH in all cases to detect *MYC*, *BCL2*, and *BCL6* rearrangements. In brief, 2-μm FFPE sections were deparaffinated by graded ethanol and digested with protease K at 37°C for 10 min, followed by the addition of 10 μl of *MYC*, *BCL2*, and *BCL6* break-apart probes (Abbott, Chicago, IL, USA). The slides were then sealed with Fixogum (Marabu, Tamm, Germany) and incubated overnight at 37°C in a hybridization chamber (Iris, Surprise, AZ, USA). Following incubation, the slides were washed with NP-40, dehydrated by graded ethanol, and stained with 5 mg/ml 4′,6-diamidino-2-phenylindole (DAPI; Beyotime, Beijing, China). The slides were independently observed by two pathologists by randomly counting 100 cells. The rearrangement was considered positive when separated signals appeared in more than 10% of counted cells. We performed IHC according to the routine process to detect the expression of CD5 (Clone: UMAB9), MYC (Clone: EP121), BCL2 (Clone: D5), BCL6 (Clone: LN22), P53 (Clone: DO-7), CD10 (UMAB235), and MUM-1 (OTI6F6). The DLBCL cases were considered to be CD5+ if more than 20% of neoplastic large B cells expressed CD5 by IHC (3). The cutoff value used for MYC was 40% (25), that for BCL2 and P53 was 50% (25), and that for BCL6, CD10, and MUM1 was 30% (26). All antibodies were obtained from Zhongshan Jinqiao (Beijing, China).

### DNA sequencing

All FFPE specimens for DNA sequencing were sectioned, and the slides were reassessed by pathologists to ensure the abundance of tumor cells (>30%) and the absence of necrosis. DNA was extracted from FFPE sections using a QIAamp DNA FFPE Tissue Kit (QIAGEN, Hilden, Germany). Briefly, 1 µg of DNA per sample was fragmented using a Bioruptor (Diagenode, Liege, Belgium). DNA libraries were constructed using the KAPA Hyper DNA Library Prep Kit (Roche, Basel, Switzerland). After library concentration and purification, hybrid selection was performed using the probes targeting 475 leukemia- and lymphoma-related genes (**Supplementary Table S1**). After the captured targets were purified and amplified, the library was normalized to 2.5 nM and sequenced as paired 150-bp reads using a HiSeq 4000 sequencing instrument (Illumina, San Diego, CA, USA).

### Bioinformatics analysis

The bioinformatics analysis was performed by GENESEEQ Technology Inc. (Nanjing, China). In brief, after sequencing, bcl2fastq v2.16.0.10 (Illumina) was used for the base calling process to generate sequence reads in FASTQ format. After quality control was applied using Trimmomatic software, high-quality reads were mapped to hg19 (GRCh37) by BWA aligner 0.7.12. Single-nucleotide variants (SNVs) and small insertions/deletions (indels) were identified by VarScan2, v2.3.9. Copy number variations (CNVs) were identified by in-house-developed software (GENSEEQ, Nanjing, China). Chromosomal instability (CIN) was determined as the average proportion of the genome harboring an aberrant copy number (log2 depth ratio >0.2 or <-0.2) as weighted on each of the 22 autosomal chromosomes (27). The tumor mutational burden (TMB) was determined by summing all base substitutions and indels in the coding region of the targeted genes, which included synonymous alterations to reduce sampling noise and excluded known driver mutations as they were overrepresented in the panel (28). For the molecular subtyping of DLBCL cases, the LymphGen classifier was applied in line with the instructions of the LymphGen website (http://llmpp.nih.gov/lymphgen/index/php). All cases were classified into the following four subtypes: MCD, EZB, BN2, and N1. The unclassified cases were considered “Other” subtype. We performed Gene Ontology (GO) analysis to annotate the biological significance based on genetic variations and Kyoto Encyclopedia of Genes and Genomes (KEGG) analysis to elucidate the key pathways among the variations. The clusterProfiler package in R was used for GO annotation and KEGG pathway analysis (29).

### Cell-of-origin analysis by Lymph2Cx assay

The COO category of each case was determined using the Lymph2Cx assay (NanoString Technologies, Seattle, WA, USA) as previously described (13). In brief, 200 ng RNA per sample was hybridized to the Lymph2Cx CodeSet under the “high sensitivity” setting on the nCounter PrepStation and then analyzed with the nCounter Analyzer (resolution: 550 field of view). The digital counts of 15 lymphoma-related mRNAs were normalized by the geometric mean of the counts of five housekeeping genes and used to calculate the linear predictor score for subgroup prediction and relative mRNA expression.

### Statistical analysis

We performed chi-square test and Fisher’s exact test to analyze the clinical, pathological, and genetic characteristics between the CD5+ and CD5- groups. The CIN, TMB, and gene expression data between the two groups were expressed as the mean ± SD, and the Student’s t-test was conducted for comparison. The Kaplan–Meier method was adopted for survival analysis. Cox proportional hazards modeling was performed for univariate and multivariate analyses to identify the factors that exert significant effects on survival. All statistical analyses were performed using SPSS16.0 software (SPSS, Chicago, IL, USA). *P* values <0.05 were considered statistically significant.

## Results

### Summary of the patients

We included 24 cases of CD5+ DLBCL and 23 cases of CD5- DLBCL in this study. The patients’ baseline features and clinicopathological parameters are presented in **Table 1**. Morphologically, the centroblastic variant comprised a large proportion in both CD5+ and CD5- DLBCL (21 of 24, 87.5% vs. 21 of 23, 91.3%, *P* > 0.999). In this study, 12.5% (3 of 24) of CD5+ DLBCL and 4.3% (1 of 23) of CD5- DLBCL were immunoblastic variant (*P* = 0.609). Only one (4.3%) CD5- DLBCL case was anaplastic variant (*P* = 0.489). Taken together, there were no significant differences in the morphology between CD5+ and CD5- DLBCL.

**Table 1:** Comparison of the baseline features and IHC results between CD5+ and CD5- DLBCL.

Characteristics	CD5+ DLBCL (N = 24)	CD5- DLBCL (N = 23)	*P* value
	n (%)	n (%)	
Baseline features			
Median age (years)	66 (43–85)	64 (29–82)	0.237
Age >60 (years)	15 (62.5)	13 (56.5)	0.676
Sex: Men	13 (54.2)	12 (52.2)	0.891
Extranodal sites (≥2)	3 (12.5)	4 (17.4)	0.638
ECOG PS (2–4)	2 (8.3)	4 (17.4)	0.297
Elevated LDH	12 (50.0)	7 (30.4)	0.172
Stage III/IV	14 (58.3)	12 (52.2)	0.671
IPI (>3)	8 (33.3)	7 (30.4)	0.831
NCCN-IPI (4–8)	8 (33.3)	8 (34.7)	0.917
BM involvement	1 (4.2)	0 (0)	0.322
CNS involvement	0 (0)	1 (4.3)	0.302
B symptoms	3 (12.5)	1 (4.3)	0.317
Bulky tumor	0 (0)	0 (0)	0.527
Hypoalbuminemia	3 (12.5)	0 (0)	0.080
HBV infection	1 (4.2)	13 (56.5)	0.276
Morphological features			
Centroblastic variant	21 (87.5)	21 (91.3)	>0.999
Immunoblastic variant	3 (12.5)	1 (4.3)	0.609
Anaplastic variant	0 (0)	1 (4.3)	0.489
IHC features			
MYC+	17 (70.8)	2 (8.7)	**<0.001**
BCL2+	14 (58.3)	11 (47.8)	0.471
BCL6+	14 (58.3)	19 (82.6)	0.069
MYC+/BCL2+	9 (37.5)	0 (0)	**0.001**
MYC+/BCL6+	10 (41.6)	1 (4.3)	**0.003**
MYC+/BCL2+/BCL6+	5 (20.8)	6 (26.1)	0.671
P53+	4 (16.6)	3 (13.0)	0.727
CD10+	9 (37.5)	8 (34.8)	0.846
MUM1+	10 (41.7)	10 (43.5)	0.900

ECGO PS, Eastern Cooperative Oncology Group performance status; LDH, lactate dehydrogenase; IPI, International Prognostic Index; NCCN, National Comprehensive Cancer Network; BM, bone marrow; CNS, central nervous system; HBV, hepatitis B virus; IHC, immunohistochemistry. The P values that <0.05 were presented in bold font.

In our study, 12.5% (3 of 24) CD5+ cases and 17.4% (4 of 23) CD5- cases had more than two extranodal involvements, with no significant difference (*P* = 0.638). The extranodal involvements were commonly observed in nasopharynx, kidney, bone marrow, and testis.

Most of CD5+ DLBCL cases (17 of 24, 70.8%) were MYC-positive compared with only two CD5- DLBCL (2 of 23, 8.7%) cases (*P* < 0.001). There was no significant difference in BCL2 expression between CD5+ and CD5- DLBCL (*P* = 0.471). A slightly lower proportion of CD5+ DLBCL cases were BCL6-positive compared with CD5- DLBCL, although the difference was not statistically significant (58.3% vs. 82.6%, *P* = 0.069). Double expressers (MYC/BCL2 or MYC/BCL6) were more common in CD5+ DLBCL (37.5% vs. 0% and 41.6% vs. 4.3%, respectively). There were no significant differences in CD10 and MUM1 expression between CD5+ and CD5- DLBCL (*P* > 0.05). The *MYC*, *BCL2*, and *BCL6* rearrangements were detected by FISH. No significant differences in *MYC* and *BCL2* rearrangements were found between CD5+ and CD5-, but more *BCL6* rearrangements were detected in CD5- DLBCL (6 of 23, 26.1%, *P* = 0.048). None of the CD5+ and CD5- DLBCL were double-hit (*MYC/BCL2* or *MYC/BCL6*) or triple-hit (*MYC/BCL2/BCL*) (**Supplementary Table S2**).

The patients in this study received standard R-CHOP/R-CHOP-like (n = 22 for CD5- DLBCL; n = 21 for CD5+ DLBCL) or R-based intensive regimens (n = 1 for CD5- DLBCL; n = 3 for CD5+ DLBCL) with two patients in each group receiving central nervous system (CNS) prophylaxis through methotrexate (MTX).

**Figure 1:**
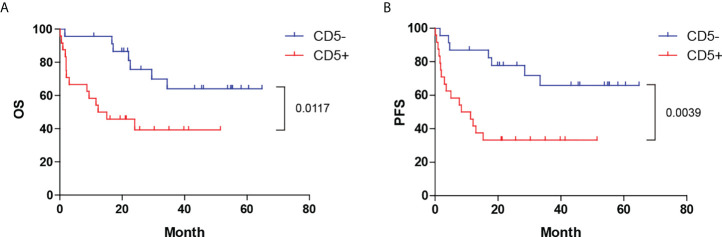
Distinct prognoses between CD5+ and CD5- DLBCL. Kaplan–Meier plot of **(A)** OS and **(B)** PFS between CD5+ and CD5- cases. OS, overall survival; PFS, progression-free survival.

Patients with CD5+ DLBCL had a significantly poorer prognosis than those with CD5- DLBCL (**Figure 1**) [median OS: 13.7 months vs. not reached, *P* = 0.0117, hazard ratio (HR): 3.082 (95% CI: 0.1320–0.7756); median progression-free survival (PFS): 9.85 months vs. not reached, *P* = 0.0039, HR: 3.474 (95% CI: 0.1242–0.6707)]. The 5-year OS and PFS for patients with CD5+ DLBCL were 40% and 33%, respectively. The complete remission (CR) rate was 33.33% (8 of 24) for patients with CD5+ DLBCL and 65.22% (15 of 23) for those with CD5- DLBCL (*P* = 0.0288).

### Genetic profiles of the CD5+ and the CD5- diffuse large B-cell lymphoma

**Figure 2:**
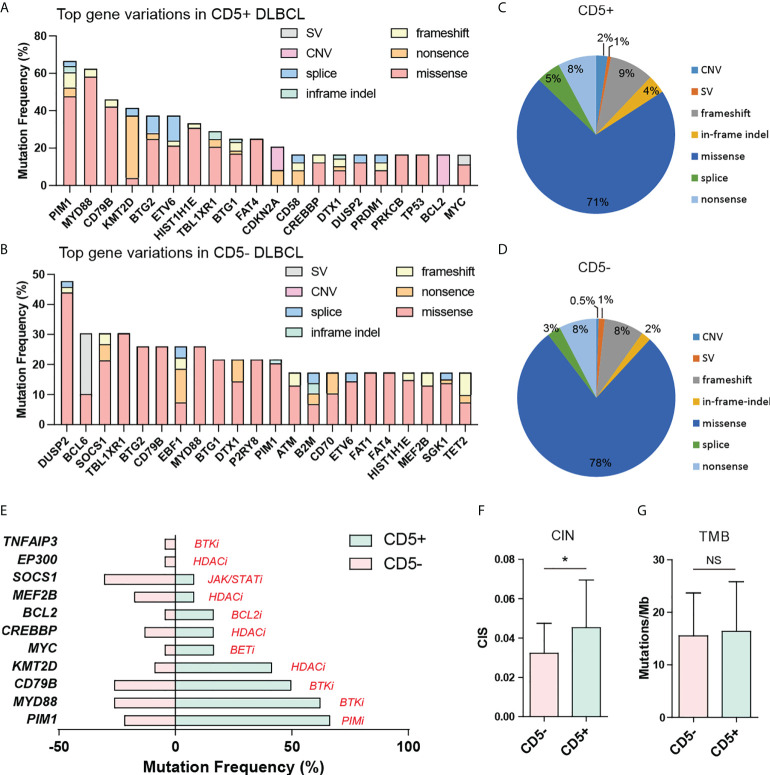
DNA-based targeted sequencing revealed the genetic disparity between CD5+ and CD5- DLBCL. The most enriched gene variations in **(A)** CD5+ DLBCL and **(B)** CD5- DLBCL showed remarkable differences. The composition of genetic variation types of **(C)** CD5+ DLBCL and **(D)** CD5- DLBCL. **(E)** Mutations with available inhibitors were more frequently shown in CD5+ DLBCL. **(F, G)** CD5+ DLBCL showed elevated CIN but similar TMB compared with CD5- DLBCL. SV, structural variation; CNV, copy number variation; CIN, chromosomal instability; TMB, tumor mutational burden; NS, not significant.

The genetic alterations, including the SNVs, small indels, splice variants, CNVs, and structural variations (SVs) of CD5+ and CD5- DLBCL cases, were determined by a lymphoma-related 475-gene panel (**Supplementary Table S1**). The *MYC*, *BCL2*, and *BCL6* gene rearrangements were simultaneously detected by DNA sequencing and FISH. The most common gene variations in CD5+ and CD5- DLBCL are shown in **Figures 2A, B**, consisting of CNVs, SVs, small in-frame/frameshift indels, and SNVs, including missense, nonsense, and splice mutations. Among the 475 genes interrogated, nearly half of the patients with CD5+ DLBCL had *PIM1*, *MYD88*, and *CD79B* variations. Other genes commonly seen variated in CD5+ DLBCL were *KMT2D*, *BTG2*, *ETV6*, *HIST1H1E*, *TBL1XR1*, *BTG1*, *FAT4*, *CDKN2A*, *CD58*, *CREBBP*, *DTX1*, *DUSP2*, *PRDM1*, *PRKCB*, *TP53*, *BCL2*, and *MYC* (**Figure 2A**). However, patients with CD5- DLBCL commonly had *DUSP2*, *BCL6, SOCS1*, and *TBLXR1* variations. Other genes commonly seen variated in CD5- DLBCL were *BTG2*, *CD79B*, *EBF1*, *MYD88*, *BTG1*, *DTX1*, *P2RY8*, *PIM1*, *ATM*, *B2M*, *CD70*, *ETV6*, *FAT1*, *FAT4*, *HIST1H1E*, *MEF2B*, *SGK1*, and *TET2* (**Figure 2B**).

CD5+ and CD5- DLBCL showed similarities in different gene variation incidences (**Figures 2C, D**), whereas CD5+ cases harbored significantly more CNVs than CD5- cases (2.4% vs. 0.5%, *P* = 0.0377). Most gene variations occurred in both CD5+ and CD5- DLBCL, with only seven genes being significantly different between the two groups (**Table 2**). CD5+ DLBCL more frequently harbored *MYD88* mutations (62.5%, n = 15, *P* = 0.0189; 13 of 15 with L265P, 1 of 15 with P245L, and 1 of 15 with Q249_K250del), *PIM1* mutations (66.7%, n = 16, *P* = 0.0032; all cases with SNVs, eight of which also had small indels), *KMT2D* mutations (41.7%, n = 10, *P* = 0.0173; all with SNVs, eight of which were nonsense mutations), and *CDKN2A* mutations (23.8%, n = 5, *P* = 0.0496; 2 of 5 with nonsense mutations and 3 of 5 with deletions). CD5- DLBCL more frequently harbored *DUSP2* mutations (47.8%, n = 11, *P* = 0.0305; 10 of 11 with SNVs and 1 of 11 with only a small indel), *TET2* mutations (17.4%, n = 4, *P* = 0.0496; 3 of 4 with SNVs, 2 of which also had small indels, and 1 of 4 with only a small indel), and *BCL6* SV (26.1%, n = 6, *P* = 0.048). The mutation of *PIM1* was the only variation correlated with OS and PFS in our cohort, which was associated with poor prognosis (**Supplementary Figures S1A, B**; *P* = 0.0214 and *P* = 0.0215, respectively). In the CD5+ group, however, the *PIM1* mutation status was not prognosis-related, although those patients with *PIM1* mutations tended to have poorer OS and PFS (**Supplementary Figures S1C, D**).

**Table 2:** Genetic variations and subtyping of CD5+/CD5- DLBCL.

Characteristics	CD5+, n = 24	CD5-, n = 23	*P*
Genetic Variations			
*MYD88*	62.5% (15/24)	26.1% (6/23)	**0.0189**
*PIM1*	66.7% (16/24)	21.7% (5/23)	**0.0032**
*KMT2D*	41.7% (10/24)	8.7% (2/23)	**0.0173**
*CDKN2A*	23.8% (5/24)	0% (0/23)	**0.0496**
*DUSP2*	16.7% (4/24)	47.8% (11/23)	**0.0305**
*TET2*	0% (0/24)	17.4% (4/23)	**0.0496**
*BCL6 SV*	4.2% (1/24)	26.1% (6/23)	**0.0480**
Genetic Subtypes			
MCD	54.17% (13/24)	13.04% (3/23)	**0.0050**
BN2	4.17% (1/24)	26.09% (5/23)	0.0971
N1	0% (0/24)	4.35% (1/23)	0.4894
EZB	4.17% (1/24)	4.35% (3/23)	0.3475
Other	37.5% (9/24)	60.87% (11/23)	0.5612

SV, structural variation. The P values that <0.05 were presented in bold font.

Numerous inhibitors targeting various pathways are available in clinical trials to treat DLBCL. CD5+, but not CD5-, cases had a higher frequency of drug-sensitive mutations (**Figure 2E**). These results indicate that this group could benefit from targeted therapies, especially from treatments based on PIM1 kinase inhibitors (PIMi), BTKi, and histone deacetylase inhibitors (HDACi). Moreover, compared with CD5- DLBCL, CD5+ DLBCL showed a higher CIN (**Figure 2F**) despite not having any significant difference in TMB (**Figure 2G**).

### Gene Ontology and Kyoto Encyclopedia of Genes and Genomes analyses of CD5+ and CD5- diffuse large B-cell lymphoma

**Figure 3:**
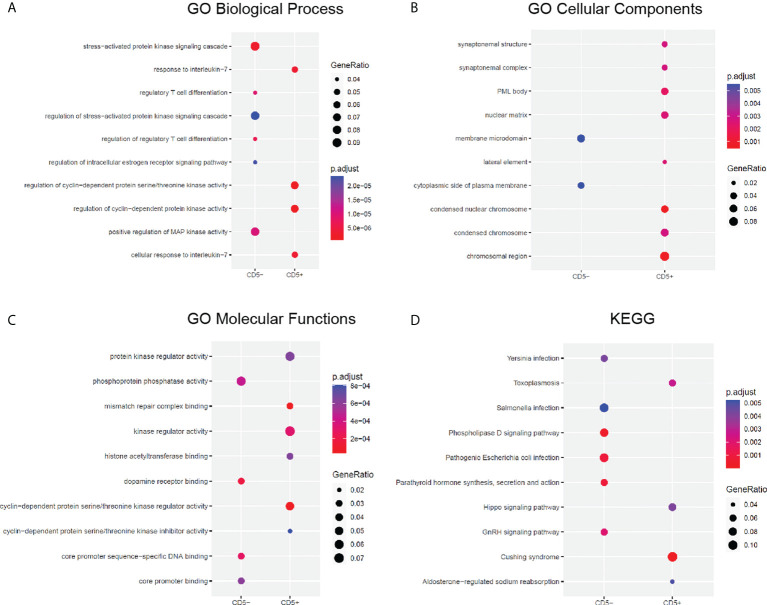
GO and KEGG analyses of CD5+ and CD5- DLBCL. The distinct GO enrichments of altered genes in CD5+ and CD5- DLBCL were ranked by significance in terms of **(A)** biological processes, **(B)** cellular components, and **(C)** molecular functions. **(D)** The distinct KEGG enrichments of altered genes in CD5+ and CD5- DLBCL were ranked by statistical significance. GO, gene ontology; KEGG, Kyoto Encyclopedia of Genes and Genomes.

GO and KEGG analyses were performed on all genes that harbored variations in CD5+ and CD5- DLBCL to clarify the potential differences in dysregulated signaling pathways and cellular components. Specifically, the unique GO and KEGG enrichments of altered genes in CD5+ and CD5- DLBCL were listed and ranked by statistical significance (**Figures 3A–C**). In CD5+ DLBCL, the distinct GO components of biological processes were “response to interleukin-7” and “regulation of cyclin-dependent protein kinase activity” (**Figure 3A**); the distinct GO components of cellular components were “chromosomal region,” “condensed nuclear chromosome,” and “nuclear matrix” (**Figure 3B**); and the distinct molecular functions were “kinase regulator activity,” “cyclin-dependent protein serine/threonine kinase regulator activity,” and “mismatch repair complex binding” (**Figure 3C**). In CD5- DLBCL, the distinct GO components of biological processes were enriched in the biological processes of “stress-activated protein kinase signaling cascade” and “positive regulation of MAP kinase activity”; the distinct GO components of cellular components were “membrane microdomain” and “cytoplasmic side of plasma membrane”; and the distinct molecular functions were “phosphoprotein phosphatase activity,” “dopamine receptor binding,” and “core promoter sequence-specific DNA binding” (**Figures 3A–C**).

In CD5+ DLBCL, the distinct KEGG enrichments were “Cushing syndrome,” “Hippo signaling pathway,” “Toxoplasmosis,” and “Aldosterone-regulated sodium reabsorption.” In CD5- DLBCL, the distinct KEGG enrichments were “Pathogenic Escherichia coli infection,” “Phospholipase D signaling pathway,” “GnRH signaling pathway,” “Parathyroid hormone synthesis, secretion and action,” “Yersinia infection,” and “Salmonella infection” (**Figure 3D**).

### Molecular subtyping of CD5+/CD5- diffuse large B-cell lymphoma

Recently, Schmitz et al. (14) reported a genetic classifier for DLBCL by subclassifying it into five categories, namely, MCD, BN2, EZB, N1, and Other. We used this classifier to perform subclassification on all CD5+ and CD5- DLBCL cases. Notably, the MCD subtype accounted for more than half of the CD5+ DLBCL cases, which was significantly higher than that observed for CD5- DLBCL (54.17% vs. 13.04%, *P* = 0.005). Furthermore, despite having no statistical significance, BN2 (26.09% vs. 4.17%, *P* > 0.05) and Other subtypes (60.87% vs. 37.5%, *P* > 0.05) accounted for the majority of CD5- DLBCL cases (**Table 2**).

We revealed whether the categorization system proposed by Schmitz et al. (14) was related to the prognosis of CD5+ and CD5- DLBCL. As shown in **Supplementary Figures S2A, B**, no significant difference in OS or PFS was observed among the five subtypes. Additionally, these subtypes showed no significant disparity in prognosis neither in CD5+ nor in CD5- group (**Supplementary Figures S2C, D**). However, the Other subtype in CD5- DLBCL presented the best prognosis, whereas the MCD subtype in CD5+ DLBCL presented the worst.

### Cell-of-origin assessment of CD5+/CD5- diffuse large B-cell lymphoma by gene expression profiling

**Figure 4:**
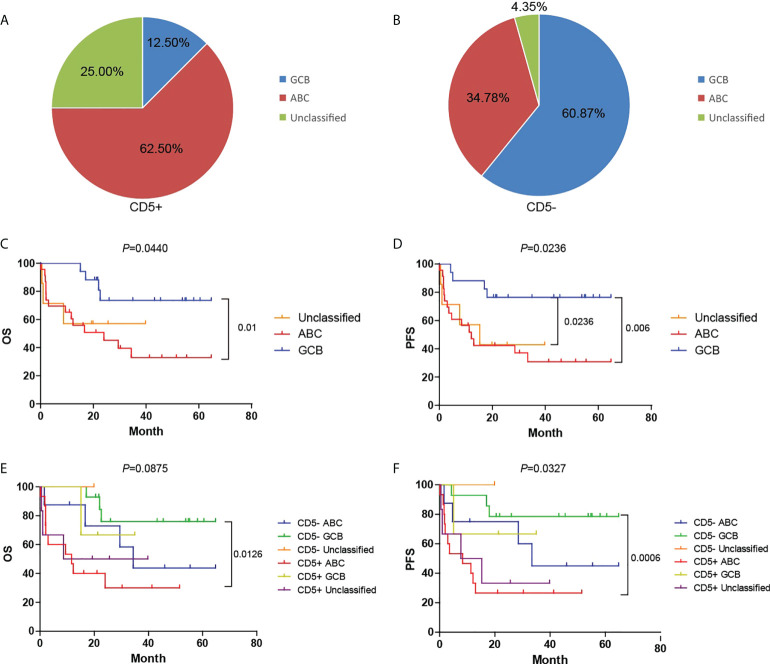
COO assessment of CD5+ and CD5- DLBCL by Lymph2Cx assay on the NanoString platform. **(A, B)** The proportion of cases classified as GCB, ABC, or unclassified in the CD5+ and CD5- groups. **(C, D)** Kaplan–Meier plot of the OS and PFS among GCB, ABC, and unclassified subtypes. **(E, F)** Kaplan–Meier plot of OS and PFS among GCB, ABC, and unclassified subtypes in CD5- and CD5+ groups separately. GCB, germinal center B cell–like; ABC, activated B cell-like.

Scott et al. (13) categorized DLBCL into three subtypes, namely, ABC, GCB, and unclassified, reflecting the theoretical COO of the neoplastic B cells, which proved to be associated with the prognosis. We determined the COO of CD5+ and CD5- DLBCL using the Lymph2Cx assay. As shown in **Figures 4A, B**, the ABC subtype accounted for 62.5% (15/24) of CD5+ DLBCL and only 34.78% (8/23) of CD5- DLBCL, whereas the GCB subtype accounted for only 12.5% (3/24) of CD5+ DLBCL and 60.87% (14/23) of CD5- DLBCL. Moreover, 25.0% (6/24) of CD5+ DLBCL and 4.35% (1/23) of CD5- DLBCL remained unclassified. These results suggested that relative to CD5- DLBCL, CD5+ DLBCL was enriched for the ABC subtype (*P* = 0.0017).

As shown in **Figures 4C, D**, the OS and PFS of DLBCL were closely associated with COO (*P* = 0.01 and *P* = 0.006, respectively). Compared with the GCB subtype, the ABC subtype showed a significantly worse prognosis; however, there was no significant difference among the COO subtypes in the CD5+ group (**Figures 4E, F**).

### Differentially expressed mRNA between CD5+ and CD5- diffuse large B-cell lymphoma

**Figure 5:**
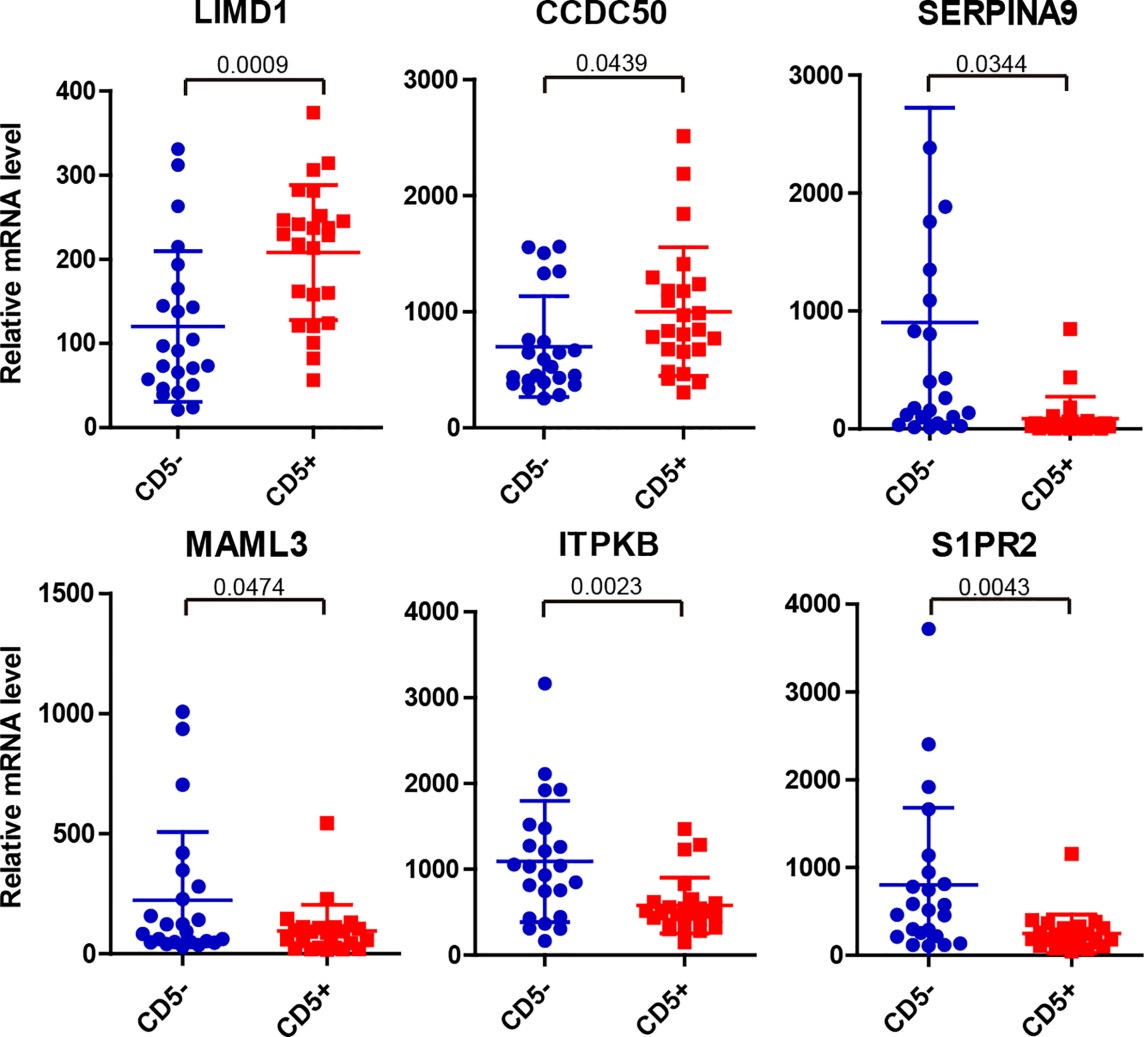
Significantly differentially expressed mRNA between CD5- and CD5+ DLBCL as detected by Lymph2Cx assay on the NanoString platform. Compared with CD5- DLBCL, *LIMD1* and *CCDC50* were upregulated whereas *SERPINA9*, *MAML3*, *ITPKB*, and *S1PR2* were downregulated in CD5+ DLBCL.

We investigated the differentially expressed mRNA between CD5+ and CD5- DLBCL by further analyzing the data generated by the Lymph2Cx assay. Compared with CD5- DLBCL, CD5+ DLBCL showed higher expression of *LIMD1* (LIM domain containing 1) and *CCDC50* (coiled-coil domain containing 50) and lower expression of *SERPINA9* (Serpin family A member 9), *MAML3* (Mastermind like transcriptional coactivator 3), *ITPKB* (Inositol-trisphosphate 3-kinase B), and *S1PR2* (Sphingosine-1-phosphate receptor 2) (**Figure 5**).

### *CYB5R2*, *MME*, and *SERPINA9* were significantly correlated with the prognosis of CD5+ diffuse large B-cell lymphoma

For each mRNA in which the expression was determined by Lymph2Cx, the median mRNA level was treated as the cutoff value to classify the cases into high- or low-expression groups. The expression level of nine mRNAs was associated with the prognosis of patients with DLBCL. We discovered that high expression of *LIMD1*, *CYB5R2* (Cytochrome b5 reductase 2), and *RAB7L1* (RAB7, member RAS oncogene family-like 1) indicated poorer OS and PFS (*P* < 0.05) (**Supplementary Figures S3A–C**, **S4A–C**). In contrast, high expression of *MME* (membrane metalloendopeptidase, also known as CD10), *SERPINA9*, *ASB13* (Ankyrin repeat and SOCS box containing 13), *MAML3*, *MYBL1* (MYB proto-oncogene like 1), and *S1PR2* indicated better outcomes (*P* < 0.05) (**Supplementary Figures S3D–I**, **S4D–I**).

**Figure 6:**
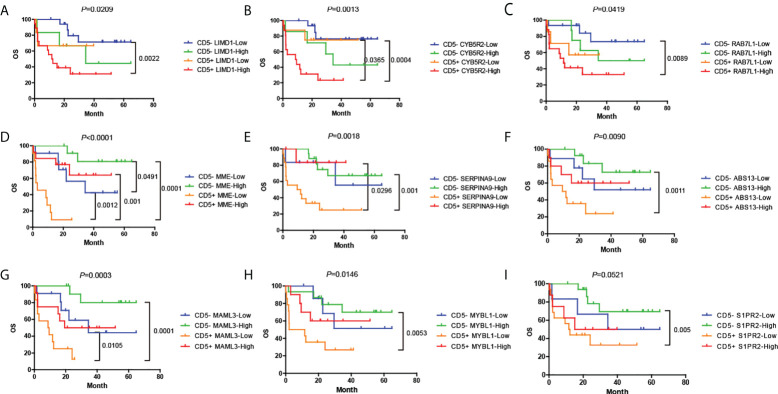
**(A–I)** Distinct prognoses between cases with high or low expression of the nine genes that correlated with OS. Cases were divided according to whether they had high or low expression of each gene by the median mRNA level, and Kaplan–Meier plots are presented for CD5- or CD5+ cases separately. OS, overall survival; PFS, progression-free survival.

We next analyzed the effects caused by the expression of these nine mRNAs on the OS and PFS of the CD5+ and CD5- groups (**Figure 6**, **Supplementary Figure S5**). Notably, three mRNAs showed a significant correlation with OS and PFS in CD5+ but not in CD5- DLBCL. In CD5+ DLBCL, low expression of *CYB5R2* predicted a more favorable OS and PFS, which was comparable to CD5-/*CYB5R2*-Low cases (*P* < 0.05) (**Figure 6B**, **Supplementary Figure S5B**), indicating that *CYB5R2* is an independent factor for unfavorable prognosis. High expression of *MME* (CD5+/*MME*-High) and *SERPINA9* (CD5+/*SERPINA9*-High) also predicted a better prognosis (*P* < 0.05) (**Figures 6D, E**; **Supplementary Figures S5D, E**) in the CD5+ but not in the CD5- group.

### Univariate and multivariate survival analyses of mRNA prognosis factors in CD5+ diffuse large B-cell lymphoma

Next, we conducted univariate and multivariate survival analyses to identify the critical factors based on the level of mRNA expression that could affect the prognosis of CD5+ DLBCL (**Table 3**). In univariate analysis, we included all clinical, immunophenotypic, genetic, and mRNA expression features. As a result, only the mRNA expression of *CYB5R2*, *MME*, and *SERPINA9* was correlated with OS (*P* = 0.037, *P* = 0.001, and *P* = 0.037, respectively), whereas that of *CYB5R2* and *MME* were correlated with PFS (*P* = 0.042 and *P* = 0.003, respectively) of CD5+ DLBCL. In multivariate analysis, we included the expression of *CYB5R2*, *MME*, *SERPINA9*, and other critical features such as the protein expression of MYC and BCL2 or BCL6, IPI, *MYC* rearrangement, and genetic subtypes. MYC/BCL2 double expression [*P =* 0.040, HR = 4.951 (95% CI: 1.078–22.741)] and ABC subtype [*P* = 0.039, HR = 7.430 (95% CI: 1.102–50.908)] were identified as independent inferior prognostic factors for OS. The *SERPINA9* mRNA expression was an independent favorable prognostic factor for OS [*P* = 0.021, HR = 0.028 (95% CI: 0.001–0.583)], and *MME* (also known as CD10) mRNA expression was an independent favorable prognostic factor for both OS [*P* = 0.002, HR = 0.038 (95% CI: 0.005–0.290)] and PFS [*P* = 0.003, HR = 0.156 (95% CI: 0.045–0.538)].

**Table 3:** Univariate and multivariate survival analyses of the mRNA expression of *CYB5R2*, *MME*, and *SERINA9* in CD5+ DLBCL.

Variables	Univariate analysis		Multivariate analysis	
	*P*	HR (95% CI)	*P*	HR (95% CI)
OS				
*CYB5R2*	**0.037**	4.368 (0.996-19.742)	0.221	5.242 (0.369-74.376)
*MME*	**0.001**	0.159 (0.047-0.547)	**0.002**	0.038 (0.005-0.290)
*SERPINA9*	**0.036**	0.152 (0.047-0.541)	**0.021**	0.028 (0.001-0.583)
MYC+/BCL2+	0.392	1.592 (0.550-4.592)	**0.040**	4.951 (1.078-22.741)
MYC+/BCL6+	0.612	0.755 (0.251-2.267)	0.707	0.567 (0.029-10.908)
*MYC* rearrangement	0.378	0.041 (0.000-50.398)	0.984	0.000 (0.000)
IPI (>3)	0.178	2.171 (0.718-6.562)	0.520	1.608 (0.378-6.845)
MCD vs. Non-MCD	0.357	1.675 (0.559-5.023)	0.746	1.434 (0.162-12.731)
ABC vs. Non-ABC	0.365	1.684 (0.526-5.391)	**0.039**	7.430 (1.102-50.908)
PFS				
*CYB5R2*	**0.042**	3.489 (0.976-12.480)	0.791	1.276 (0.211-7.729)
*SERPINA9*	0.053	0.260 (0.093-0.0728)	/	
*MME*	**0.003**	0.222 (0.076-0.649)	**0.003**	0.156 (0.045-0.538))
MYC+/BCL2+	0.295	1.698 (0.630-4.579)	0.200	1.980 (0.697-5.625)
MYC+/BCL6+	0.699	0.819 (0.296-2.268)	0.851	0.864 (0.315-6.361)
*MYC* rearrangement	0.420	0.433 (0.057-3.307)	0.154	0.207 (0.024-1.801)
IPI>3	0.121	2.230 (0.810-6.139)	0.650	1.417 (0.515-12.094)
MCD vs. Non-MCD	0.405	1.544 (0.556-4.294)	0.523	1.476 (0.447-4.870)
ABC vs. Non-ABC	0.410	1.564 (0.540-4.531)	0.499	1.580 (0.420-5.949)

OS, overall survival; PFS, progression-free survival; IPI, International Prognostic Index; ABC, activated B-cell like. The P values that <0.05 were presented in bold font.

## Discussion

As a group of DLBCLs with distinct clinical and pathological characteristics, CD5+ DLBCL accounts for 5%–20% of all DLBCL cases. Compared with patients with CD5- DLBCL, those with CD5+ DLBCL tend to have a poorer prognosis, with a 5-year OS of only 35%. In addition, patients with CD5+ DLBCL are more likely to suffer recurrence under the current R-CHOP regime (22, 30). In this study, we performed DNA sequencing and Lymph2Cx assay to elucidate the disparity and prognostic value of the molecular characteristics shown by CD5+ and CD5- DLBCL.

CD5 expression was identified as a risk factor for the poor outcomes of DLBCL. In our study, the 5-year OS and PFS were 40% and 33% in CD5+ DLBCL, respectively, which is highly consistent with previous studies (31–35). We also evaluated other prognosis-related factors and found that MYC was more commonly expressed in CD5+ DLBCL (17/24, *P* < 0.05), while we did not find a significant difference in BCL-2 and BCL-6 expression between CD5+ and CD5- DLBCL (*P* > 0.05). Co-expression of MYC/BCL6 and MYC/BCL2 was common in patients with CD5+ DLBCL, which is consistent with the findings of previous studies (36). Since few studies suggest the association between CD5 expression and MYC expression, further studies are warranted to clarify the mechanism of MYC dysregulation in CD5+ DLBCL.

Genetic variations, including SNVs, CNVs, and SVs, have been discovered in DLBCL and have been proven to be risk stratification markers of prognostic value (37, 38). Recently, the extensive high-throughput sequencing of gene variations in DLBCL boiled down to several promising classifiers to predict prognosis and assist treatment decision-making (14, 16, 18, 39). *MYD88* mutation, which was typically presented as *MYD88^L265P^*, was one of the most common mutations in ABC DLBCL and indicated a poor prognosis (40). *PIM1* mutation, also commonly observed in ABC DLBCL, was reported to reduce the sensitivity to ibrutinib treatment (41) and was associated with poor prognosis (42). In our study, *MYD88* and *PIM1* mutations occurred predominantly in CD5+ DLBCL. Although patients with *PIM1* mutation had significantly poorer outcomes, we did not observe any significant differences in the CD5+ group. Moreover, Schmitz et al. (14) reported that *MYD88* and *PIM1* mutations were enriched in the MCD subtype. The C5 subtype proposed by Chapuy et al. (16) and the MYD88 subtype proposed by Lacy et al. (39) also were characterized by *MYD88* and *PIM1* mutations. As expected, more than half of the CD5+ cases were classified as the MCD subtype (*P* = 0.005), but the CD5- cases did not trend toward a particular subtype. Despite not finding any prognostic differences found between various subtypes in CD5+ cases, the MCD group appeared to show more adverse outcomes than the non-MCD group.

*KMT2D*, an epigenetic regulator that mediates germinal center B-cell development under physiological conditions and leads to lymphomagenesis when disrupted (43), was shown to be mutated in CD5+ DLBCL, mostly with nonsense mutations. As another epigenetic regulator and proto-oncogene, *TET2* was shown to be predominantly mutated in CD5- DLBCL, suggesting a different mechanism of tumorigenesis between these two groups. *CDKN2A* mutation was also common in CD5+ DLBCL with two cases of nonsense mutations and three cases of deletions, all of which were considered as loss-of-function variations. *CDKN2A* is a canonical tumor suppressor that regulates cell cycle, the loss of which may lead to perturbed chromosomal stability and poor prognosis (44). Consistently, we found a significant increase in CIN in CD5+ DLBCL, which has been reported to indicate a worse outcome in DLBCL (45). However, the reason for the observed CIN in CD5+ DLBCL remains unclear.

Patients with CD5+ DLBCL usually have a high risk of CNS involvement/relapse (16). In our previously published data with a larger sample (24), CNS involvement occurred in 16.4% (32/195) of CD5+ DLBCL cases. Moreover, *MYD88*, *CD79B*, and *PIM1* mutations were commonly found in primary CNS lymphomas and DLBCLs with CNS relapse (16). However, only one CD5- patient with ABC and BN2 subtypes experienced CNS relapse without CNS prophylaxis in this study, and no significant differences were observed between CD5- and CD5+ DLBCL, which probably was due to the small sample size.

**Figure 7:**
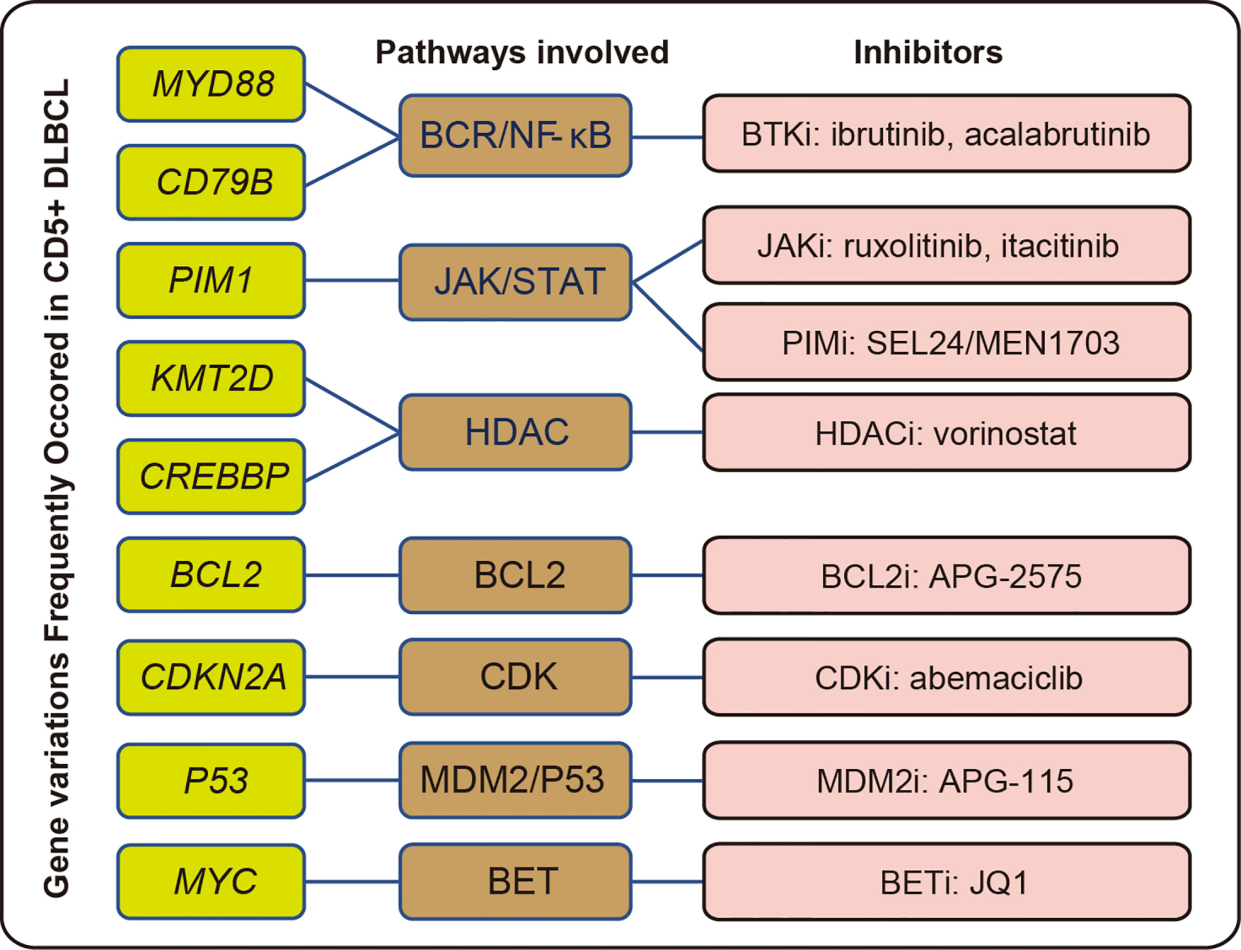
Summary of common gene variations with potentially related pathways and inhibitors in CD5+ DLBCL.

Recently, alterations in genes and their expression have been shown to be critical for precision treatment. Small-molecule inhibitors targeting various kinases were widely used in DLBCL, including PIMi, HDACi, and BTKi (46–48). It has been reported that patients with DLBCL harboring *MYD88* and/or *CD79B* mutations are more sensitive to BTKi (49, 50). More recently, PIMi was reported to enhance the efficacy of CD20 antibodies by targeting *MYC* transcription (51). Inhibitors targeting epigenetic alterations have been widely studied, and the application of HDACi was considered to be a feasible solution for treating patients with *KMT2D* alterations (52). We demonstrated a significant enrichment in *MYD88*, *CD79B*, *PIM1*, and *KMT2D* mutations in CD5+ DLBCL (**Figure 2E**), which implied that applying the corresponding inhibitors might improve the treatment outcome of this group. The gene variations, potentially involved pathways, and corresponding inhibitors are summarized in **Figure 7** (53–58).

The NanoString platform not only facilitates the routine gene expression-based COO classification of DLBCL but also allows this analysis to be conducted on FFPE tissues (59). In this study, we performed a Lymph2Cx assay to detect the mRNA expression of 15 lymphoma-related genes on the NanoString platform for COO analysis. Compared with other reports in which COO was analyzed by RNA array (60, 61), 62.5% of CD5+ DLBCL and only 34.78% of CD5- DLBCL were classified into ABC subtype. The DLBCL classified as ABC subtype indeed showed lower OS and PFS, as has been reported previously (62). In CD5+ cases, the ABC subgroup manifested a more unfavorable prognosis in OS and PFS than the GCB subgroup, although this was not statistically significant, which again may have been due to the relatively small sample size.

In our study, six of the 15 analyzed mRNAs were differentially expressed between CD5+ and CD5- cases, among which *LIMD1* and *CCDC50* were significantly upregulated in the CD5+ group. These two genes acted as the regulators of NF-κB and were considered to be markers for ABC DLBCL (63–65). The other four differentially expressed genes, *SERPINA9*, *MAML3*, *ITPKB*, and *S1PR2*, all of which were suggested to be overexpressed in GBC DLBCL (59), were downregulated in CD5+ DLBCL. We also performed a survival analysis on the mRNA level of these 15 genes, in which the median mRNA level of all cases was treated as the cutoff value, and the cases were divided into high- and low-expression groups. As a result, nine of the 15 genes showed significant correlations in terms of OS and PFS. However, only three genes exerted significant effects on the prognosis in the CD5+ group. The CD5+ cases with low expression of *CYB5R2* and high expression of *MME* and *SERPINA9* presented a more favorable prognosis, indicating that even in the CD5+ group, the expression of certain markers might indicate a better prognosis. We next conducted univariate and multivariate survival analyses. In multivariate analysis, the MYC/BCL2 double expression, ABC subtype, and the mRNA expression of *SERPINA9* showed a significant correlation with OS, while the mRNA expression of *MME* showed a significant correlation with both OS and PFS.

*CYB5R2* is considered to be a tumor suppressor in prostate cancer and nasopharyngeal cancer (66–68). Although its function is unclear in DLBCL, it was applied as an ABC marker in the Lymph2Cx assay (13). Low expression of *CYB5R2* showed a significant correlation with a better prognosis in CD5+ DLBCL in the univariate but not in the multivariate survival analysis, indicating that the favorable prognosis of *CYB5R2*-Low cases was probably attributed to other factors, such as the COO. *SERPINA9* is a protease inhibitor whose expression is restricted to germinal center B cells and lymphoid malignancies with germinal center B-cell maturation and is associated with a good prognosis in DLBCLs (69). *MME*, also known as CD10, is a canonical GCB DLBCL marker, which was applied for COO classification by Hans et al. (26). Despite some opposing reports (70, 71), CD10+ cases commonly have shown favorable prognosis in DLBCL (26, 72). In multivariate survival analysis, the expressions of *MME* and *SERPINA9* were prognostic factors independent of COO, suggesting that these genes have other effects on CD5+ DLBCL, as opposed to simply representing markers for the GCB subtype. In our cohort, the low expression of *MME* and *SERPINA9* presented an extremely poor prognosis in the CD5+ group; however, in *MME*-High and *SERPINA*-High cases, CD5 expression showed no effect on the OS and PFS, which indicated that *MME* and *SERPINA9* are protective factors and prognosis markers that are independent of CD5.

This study has some limitations that warrant discussion. First, the sample size was small, which reduced the robustness of our method. Second, we only used gene expression to predict the prognosis of a patient, and recent studies have suggested that pathological images (73, 74) and other types of molecular data are also critical for prognosis. Therefore, in the future, it may be necessary to improve the performance of prognosis analysis by introducing multi-omics study, image analysis, and machine learning. Finally, the classification model used in this study was relatively simple, focusing only on LymphGen algorithms. Indeed, many studies have proposed different classifiers based on respective experimental data, such as the Dana-Farber Cancer Institute (DFCI) classification put forward by Chapuy et al. (16) and the Lymphoma Study Association (LYSA) classification proposed by Dubois et al. (17). Using our data generated by DNA-targeted sequencing, however, we were unable to perform a similar classification through DFCI and LYSA classifiers, which require whole-exome sequencing or chromosome arm copy number analysis. Although several influential studies have concluded that the subtypes of LymphGen and DFCI have similar characteristics in terms of COO, genetic hallmarks, and prognosis (39, 75), more research is necessary to clarify the molecular characteristics of CD5+ DLBCL using different classification tools.

In conclusion, in this study, we characterized the genetic profile of CD5+ DLBCL by *PIM1*, *MYD88*, and *CD79B* mutations, with MCD and ABC subtypes commonly observed. MYC/BCL2 double expression, ABC subtype, and mRNA expression of *SERPINA9* and *MME* were independently predictive of the prognosis of CD5+ DLBCL.

## Data availability statement

The data presented in the study are deposited in the NCBI BioProject repository, accession number PRJNA838469, (https://www.ncbi.nlm.nih.gov/bioproject/PRJNA838469).

## Author contributions

DM and YHM are co-first authors and contributed to the experiment conduction, data analysis and manuscript preparation. YYM, JL, YG, and NL contributed to the collection of pathological data. CX contributed to the data analysis and manuscript preparation. HL and WS are co-corresponding authors and contributed to the project design. All authors contributed to the article and approved the submitted version.

## Funding

This project was supported by the Key Research and Development Project of Xuzhou (KC21307).

## Conflict of interest

The authors declare that the research was conducted in the absence of any commercial or financial relationships that could be construed as a potential conflict of interest.

## Publisher’s note

All claims expressed in this article are solely those of the authors and do not necessarily represent those of their affiliated organizations, or those of the publisher, the editors and the reviewers. Any product that may be evaluated in this article, or claim that may be made by its manufacturer, is not guaranteed or endorsed by the publisher.

## Supplementary material

Supplementary Figure 1 Distinct prognoses between cases with (*PIM1*+) and without (*PIM1*-) *PIM1* mutation. **(A, B)** Kaplan-Meier plots of OS and PFS between *PIM1*+ and *PIM1*- cases. **(C, D)** Kaplan-Meier plots of OS and PFS between *PIM1*+ and *PIM1*- cases in CD5+ and CD5- groups separately.

Supplementary Figure 2 Distinct prognoses between cases with different genetic subtypes by LymphGen. **(A, B)** Kaplan-Meier plots of OS and PFS among BN2, EZB, MCD, N1, and Other subtypes. **(C, D)** Kaplan-Meier plots of OS and PFS among BN2, EZB, MCD, N1, and Other subtypes in the CD5- group, or between MCD and non-MCD in the CD5+ group.

Supplementary Figure 3 mRNA expression of nine genes showed a significant correlation with OS, including **(A)** *LIMD1*, **(B)** *CYB5R2*, **(C)** *RAB7L1*, **(D)** *MME*, **(E)** *SERPINA9*, **(F)** *ABS13*, **(G)** *MAML3*, **(H)** *MYBL1*, and **(I)** *S1PR2*.

Supplementary Figure 4 mRNA expression of nine genes showed a significant correlation with PFS, including **(A)** *LIMD1*, **(B)** *CYB5R2*, **(C)** *RAB7L1*, **(D)** *MME*, **(E)** *SERPINA9*, **(F)** *ABS13*, **(G)** *MAML3*, **(H)** *MYBL1*, and (I) *S1PR2*.

Supplementary Figure 5
**(A–I)** Distinct prognoses between cases with high or low mRNA expression of nine genes that correlated with PFS. Cases were divided according to whether each gene had a high or low expression by the median mRNA levels, and Kaplan-Meier plots are presented for CD5- or CD5+ cases separately.
