# Supplementary figures and images for: Molecular subtyping of CD5+ diffuse large B-cell lymphoma based on DNA-targeted sequencing and Lymph2Cx
Source: Front Oncol. 2022 Aug 23;12:941347. doi: 10.3389/fonc.2022.941347 (PMC9445310; doi:10.3389/fonc.2022.941347)

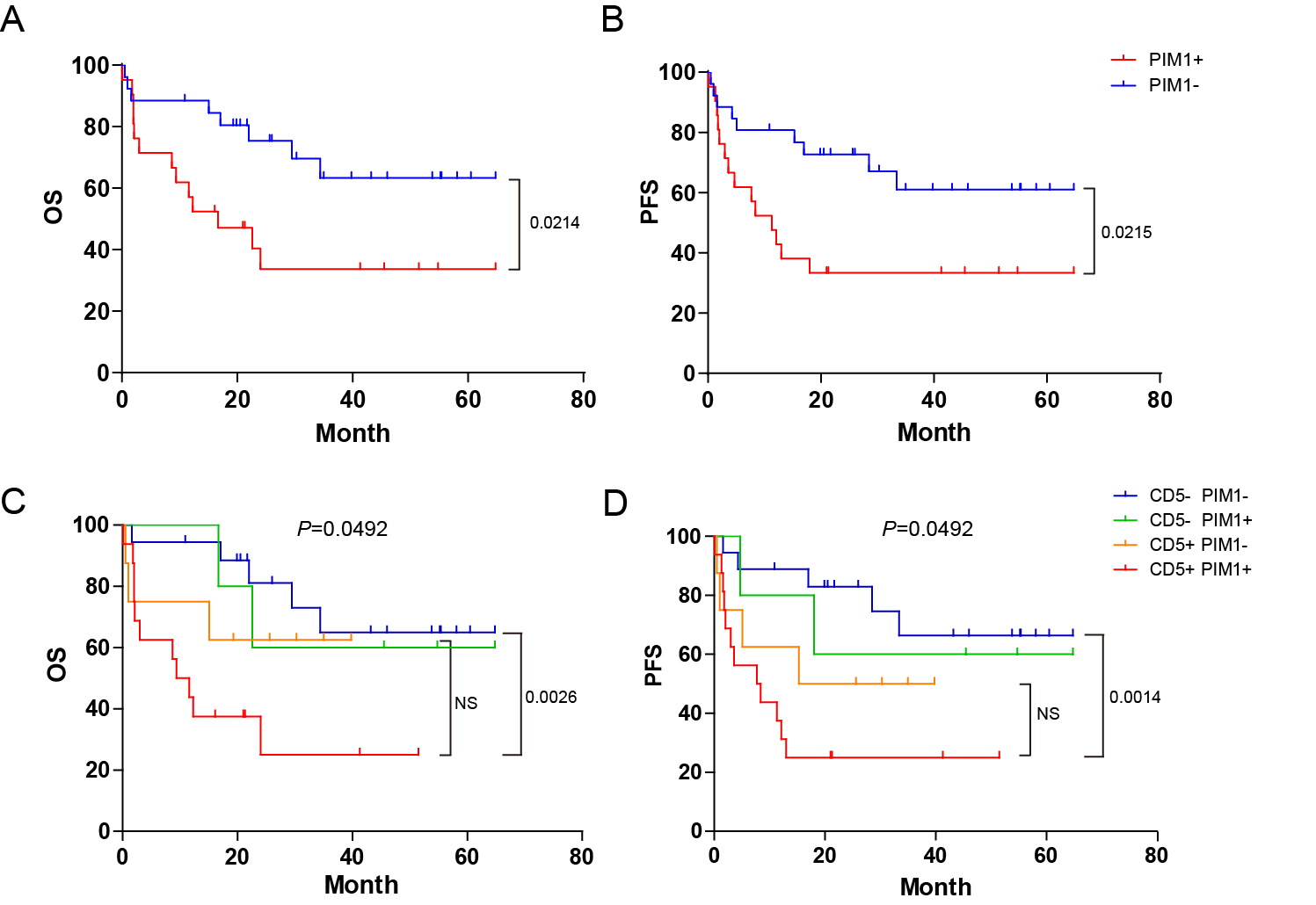

Supplement: Supplementary Figure 1 — Distinct prognoses between cases with (PIM1+) and without (PIM1-) PIM1 mutation. (A, B) Kaplan-Meier plots of OS and PFS between PIM1+ and PIM1- cases. (C, D) Kaplan-Meier plots of OS and PFS between PIM1+ and PIM1- cases in CD5+ and CD5- groups separately. [file Image_1.jpeg]

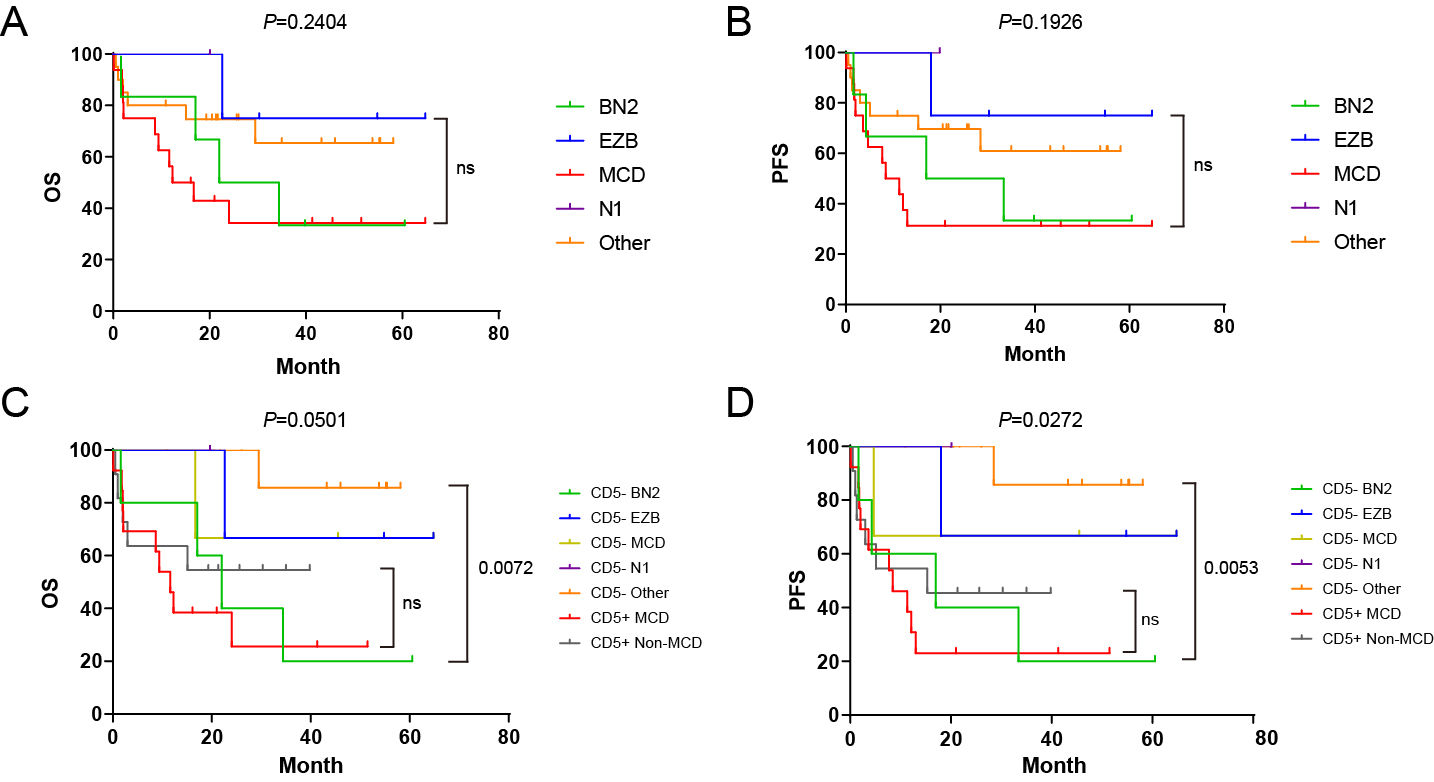

Supplement: Supplementary Figure 2 — Distinct prognoses between cases with different genetic subtypes by LymphGen. (A, B) Kaplan-Meier plots of OS and PFS among BN2, EZB, MCD, N1, and Other subtypes. (C, D) Kaplan-Meier plots of OS and PFS among BN2, EZB, MCD, N1, and Other subtypes in the CD5- group, or between MCD and non-MCD in the CD5+ group. [file Image_2.jpeg]

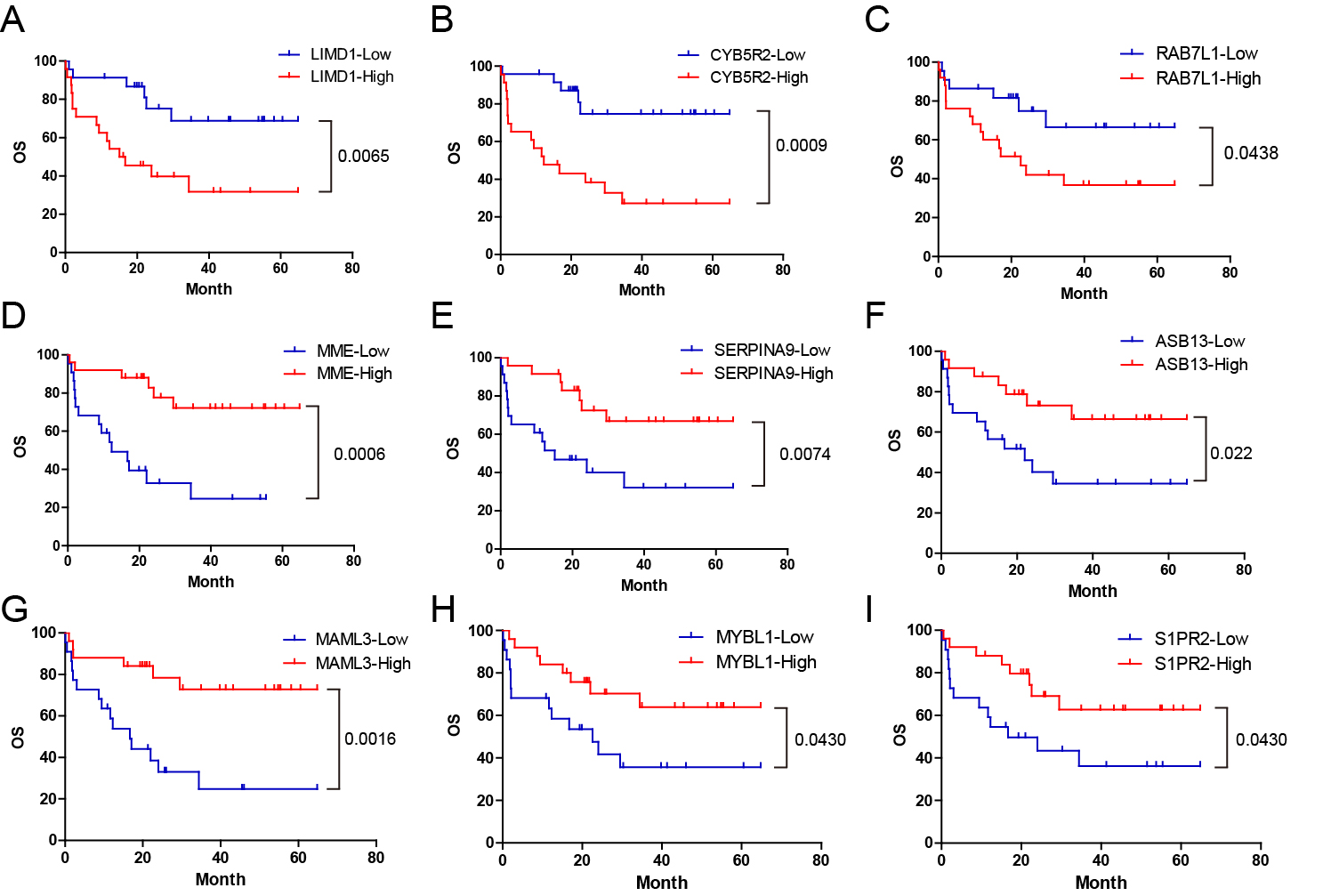

Supplement: Supplementary Figure 3 — mRNA expression of nine genes showed a significant correlation with OS, including (A) LIMD1, (B) CYB5R2, (C) RAB7L1, (D) MME, (E) SERPINA9, (F) ABS13, (G) MAML3, (H) MYBL1, and (I) S1PR2. [file Image_3.jpeg]

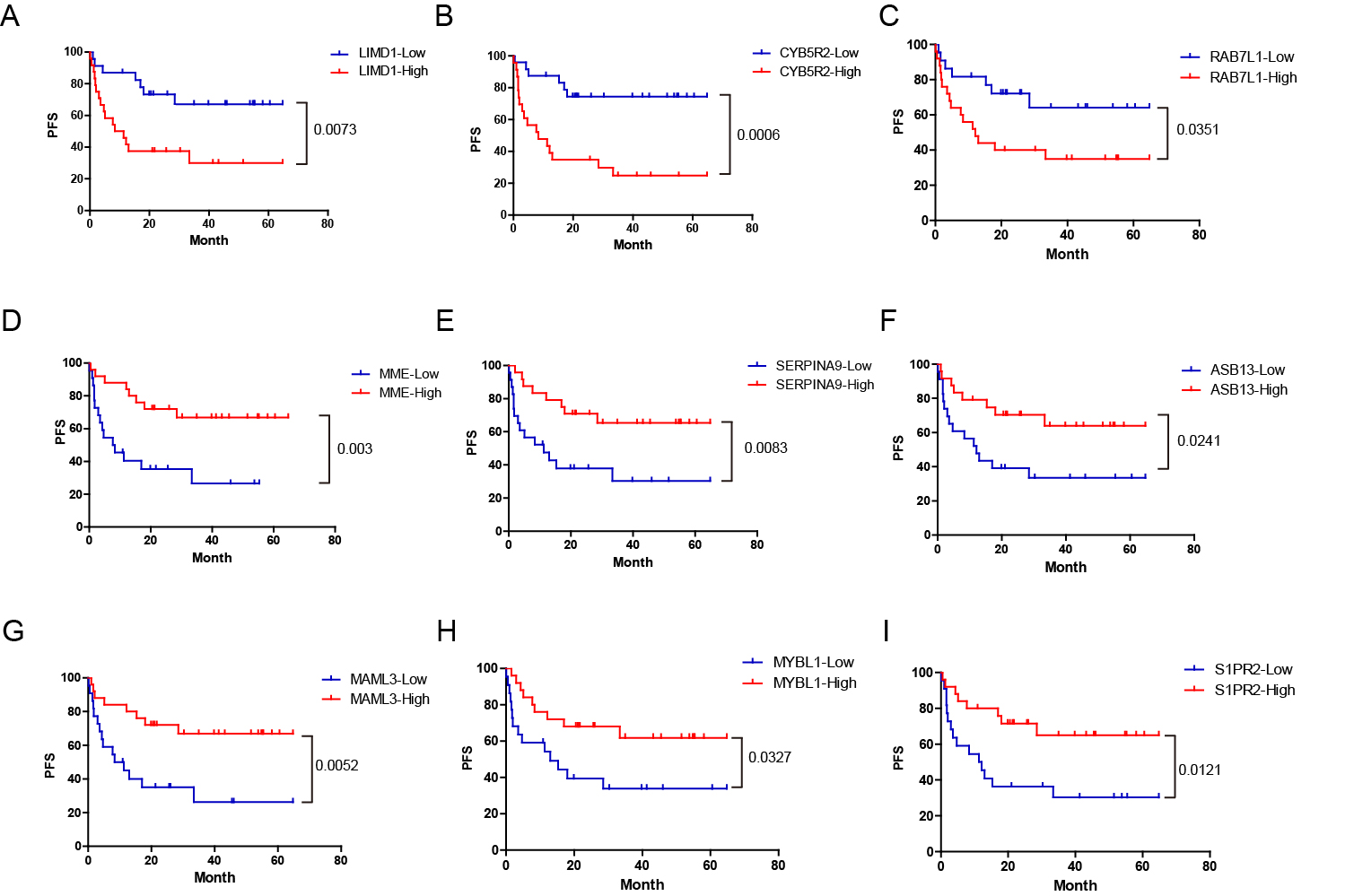

Supplement: Supplementary Figure 4 — mRNA expression of nine genes showed a significant correlation with PFS, including (A) LIMD1, (B) CYB5R2, (C) RAB7L1, (D) MME, (E) SERPINA9, (F) ABS13, (G) MAML3, (H) MYBL1, and (I) S1PR2. [file Image_4.jpeg]

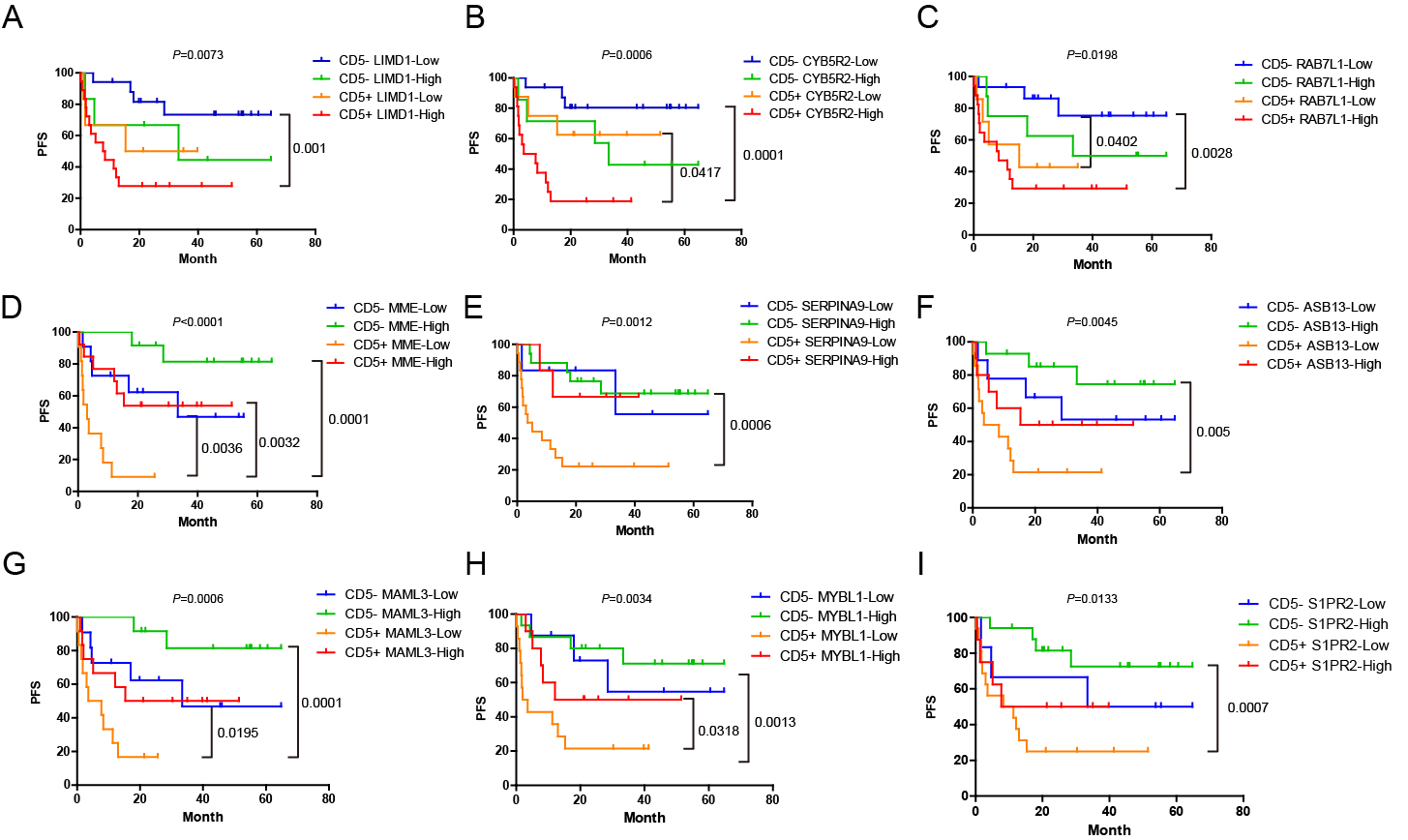

Supplement: Supplementary Figure 5 — (A–I) Distinct prognoses between cases with high or low mRNA expression of nine genes that correlated with PFS. Cases were divided according to whether each gene had a high or low expression by the median mRNA levels, and Kaplan-Meier plots are presented for CD5- or CD5+ cases separately. [file Image_5.jpeg]
