# Supplementary material for: Molecular subtyping of CD5+ diffuse large B-cell lymphoma based on DNA-targeted sequencing and Lymph2Cx
Source: Front Oncol. 2022 Aug 23;12:941347. doi: 10.3389/fonc.2022.941347 (PMC9445310; doi:10.3389/fonc.2022.941347)
Supplement: Supplementary file 7 [file Table_2.docx]

| **Rearrangement features** | **CD5+DLBCL(N=24)**  **n(%)** | **CD5- DLBCL(N=23)**  **n(%)** | ***P* value** |
| --- | --- | --- | --- |
| *MYC+* | 2(8.3) | 0(0) | 0.489 |
| *BCL2+* | 0(0) | 1(4.3) | 0.489 |
| *BCL6 +* | 1(4.2) | 6(26.1) | **0.048** |
| *MYC+*/*BCL2*+ or *BCL6*+ | 0(0) | 0(0) | - |
| *MYC*+/*BCL2*+/*BCL6*+ | 0(0) | 0(0) |  |

Supplementary Table 2. Rearrangement results between CD5+ and CD5- DLBCL
